# Supplementary material for: Reduced SKP2 Expression Adversely Impacts Genome Stability and Promotes Cellular Transformation in Colonic Epithelial Cells
Source: Cells. 2022 Nov 22;11(23):3731. doi: 10.3390/cells11233731 (PMC9738323; doi:10.3390/cells11233731)
Supplement: Supplementary file 1 [file cells-11-03731-s001.zip › cells-1965065-supplementary.pdf]

SUPPLEMENTARY INFORMATION

SUPPLEMENTARY FIGURES

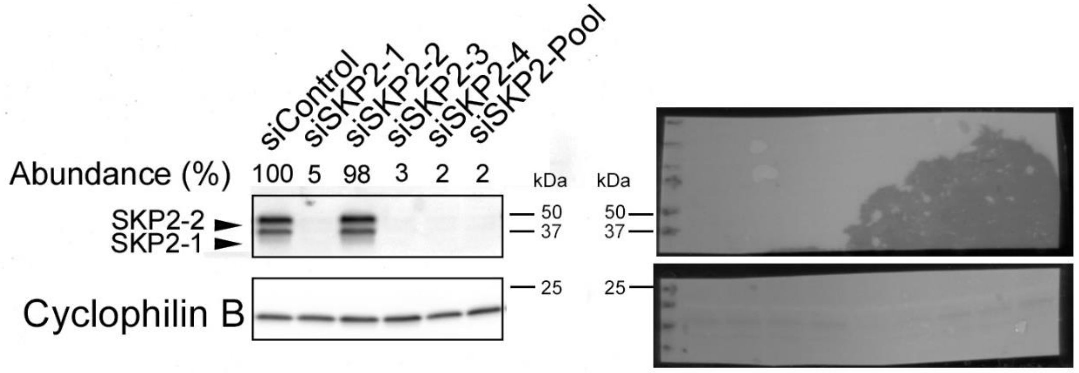

	SKP2	Cyclophilin B	Ratio S/C	Norm. ratio
siControl	84.01	63.39	1.33	100.00
siSKP2-1	4.41	63.24	0.07	5.26
siSKP2-2	93.32	71.53	1.30	98.45
siSKP2-3	3.17	72.54	0.04	3.29
siSKP2-4	1.91	62.48	0.03	2.30
siSKP2-Pool	2.08	69.36	0.03	2.26

Figure S1: Raw data for western blot in Figure 3.
Chemiluminescence (left) and visible light (right) images of the SKP2 and Cyclophilin B western blots shown in Figure 3A. Note that two bands representing the two SKP2 isoforms (SKP2-1 and SKP2-2; left labeling) are visible. Black rectangles highlight the cropped regions presented in Figure 3A. Densitometry analyses for SKP2 and Cyclophilin B were performed using Image J and are indicated. The ratio of SKP2/Cyclophilin B is shown for each lane, as are the normalized ratios, which are presented relative to the siControl.

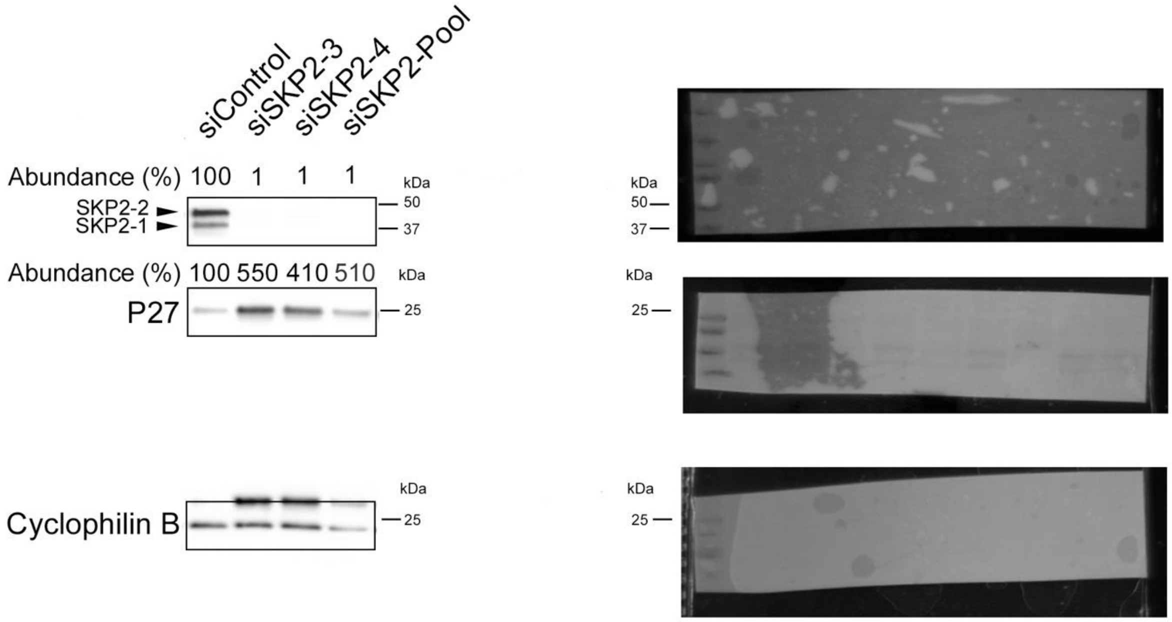

				Normalized Abundance		Relative Abundance	
	**SKP2**	**P27**	**Cyclophilin B**	**SKP2/CycloB**	**P27/CycloB**	**SKP2/CycloB**	**P27/CycloB**
**siControl**	35515.853	1985.406	32585.288	1.089935219	0.060929521	100.0	100.0
**siSKP2-3**	407.435	11818.811	35248.652	0.011558882	0.335298241	1.1	550.3
**siSKP2-4**	479.607	9689.104	38722.966	0.012385596	0.250215957	1.1	410.7
**siSKP2-Pool**	101.155	3615.719	11624.752	0.008701691	0.311036227	0.8	510.5

Figure S2. Semi-quantitative western blot depicting SKP2 and P27 abundance following *SKP2* silencing in HCT116 cells.

Note that two bands representing the two SKP2 isoforms (SKP2-1 and SKP2-2; left labeling) are visible. Semi-quantitative analyses were performed whereby SKP2 and P27 abundance were first normalized to the respective loading control (Cyclophilin B) and are presented relative to siControl (100%). Densitometry analyses for SKP2, P27 and Cyclophilin B were performed using Fiji and are indicated. The relative abundance (%) of SKP2 and P27 are presented above each respective lane.

A

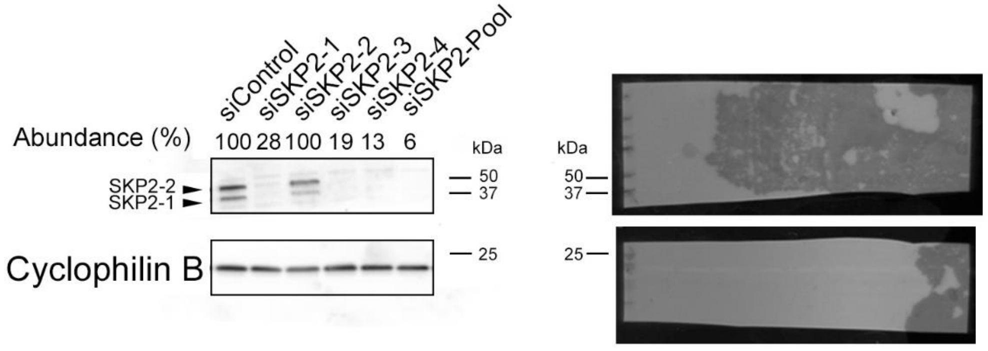

	SKP2	Cyclophilin B	Ratio S/C	Norm. ratio
**siControl**	69.14	79.82	0.87	100.00
**siSKP2-1**	15.90	65.83	0.24	27.88
**siSKP2-2**	51.13	59.27	0.86	99.59
**siSKP2-3**	12.86	76.83	0.17	19.33
**siSKP2-4**	9.03	78.78	0.11	13.24
**siSKP2-Pool**	3.70	74.03	0.05	5.77

B

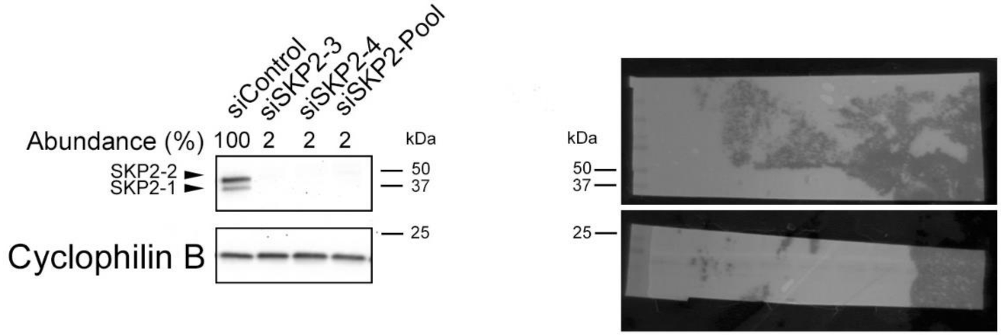

	SKP2	Cyclophilin B	Ratio S/C	Norm. ratio
**siControl**	51.05	60.09	0.85	100.00
**siSKP2-3**	0.91	64.32	0.01	1.67
**siSKP2-4**	0.92	70.23	0.01	1.55
**siSKP2-Pool**	1.11	59.92	0.02	2.17

Figure S3: Raw data for western blots in Figure 5.
Chemiluminescence (left) and visible light (right) images of the SKP2 and Cyclophilin B western blots shown in Figure 5A including 1CT ***(A)*** and A1309 ***(B)***. Black rectangles highlight the cropped regions shown in Figure 5A. Densitometry analyses for SKP2 and Cyclophilin B were performed using Image J and are indicated. The ratio of SKP2/Cyclophilin B is shown for each lane, as are the normalized ratios, which are presented relative to the siControl.

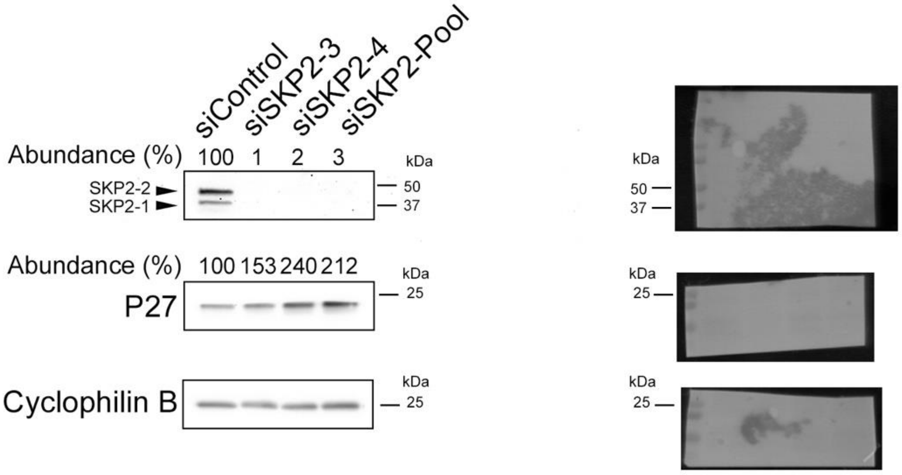

				Normalized Abundance		Relative Abundance	
	**SKP2**	**P27**	**Cyclophilin B**	**SKP2/CycloB**	**P27/CycloB**	**SKP2/CycloB**	**P27/CycloB**
**siControl**	35129.966	12934.125	33745.681	1.041021101	0.383282382	100.0	100.0
**siSKP2-3**	297.163	15544.974	26518.995	0.011205666	0.586182621	1.1	152.9
**sSKP2-4**	662.456	27186.388	29550.51	0.022417752	0.919997252	2.2	240.0
**siSKP2-Pool**	1211.912	34013.38	41803.874	0.028990423	0.813641817	2.8	212.3

Figure S4. Semi-quantitative western blot depicting SKP2 and P27 abundance following *SKP2* silencing in A1309 cells.

Note that two bands representing the two SKP2 isoforms (SKP2-1 and SKP2-2; left labeling) are visible. Semi-quantitative analyses were performed whereby SKP2 and P27 abundance were first normalized to the respective loading control (Cyclophilin B) and are presented relative to siControl (100%). Densitometry analyses for SKP2, P27 and Cyclophilin B were performed using Fiji and are indicated. The relative abundance (%) of SKP2 and P27 are presented above each respective lane.

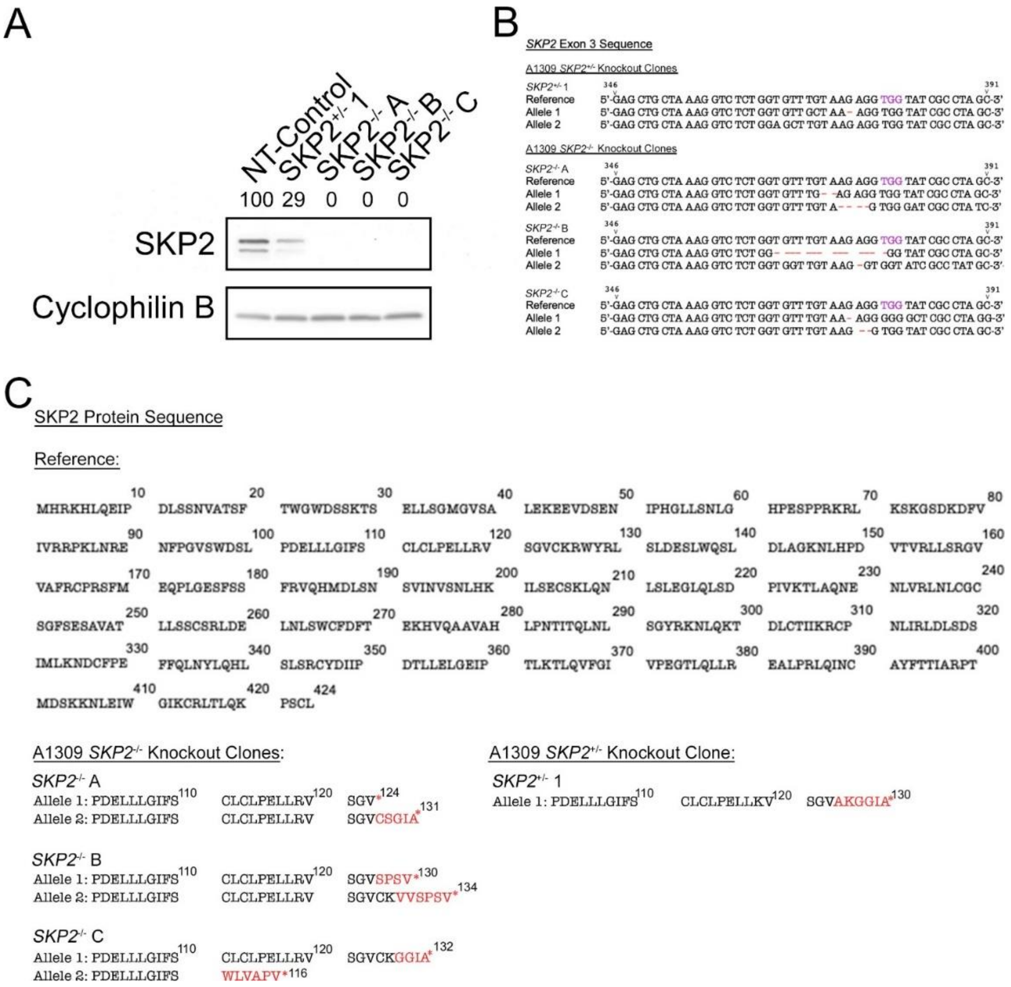

Figure S5. DNA Sequencing Analyses Identifies *SKP2*$^{+/-}$ and *SKP2*$^{-/-}$ Knockout Clones in A1309.

(A) Semi-quantitative western blots presenting the A1309 clones pursued in long-term CIN assays (p20). Decreased *SKP2* expression is presented relative to NT-Control (100%) with Cyclophilin B serving as the loading control. ***(B)*** DNA sequencing results for exon 3 of *SKP2* in one *SKP2*$^{+/-}$ clone and three *SKP2*$^{-/-}$ clones relative to the reference sequence (NM_005983.4). Numbers indicate nucleotide position in the *SKP2* cDNA. PAM (purple font); protospacer adjacent motif. ***(C)*** Predicted amino acid alterations corresponding to DNA sequenced verified allele-specific edits following CRISPR/Cas9-mediated editing of *SKP2*. Numbers indicate amino acid position relative to the reference sequence (NM_005974.2; top sequence). Red font identifies the divergent amino acids stemming from the CRISPR/Cas9 edits. All frameshift mutations are predicted to incorporate premature stop codons (*) that are expected to induce nonsense-mediated mRNA decay.

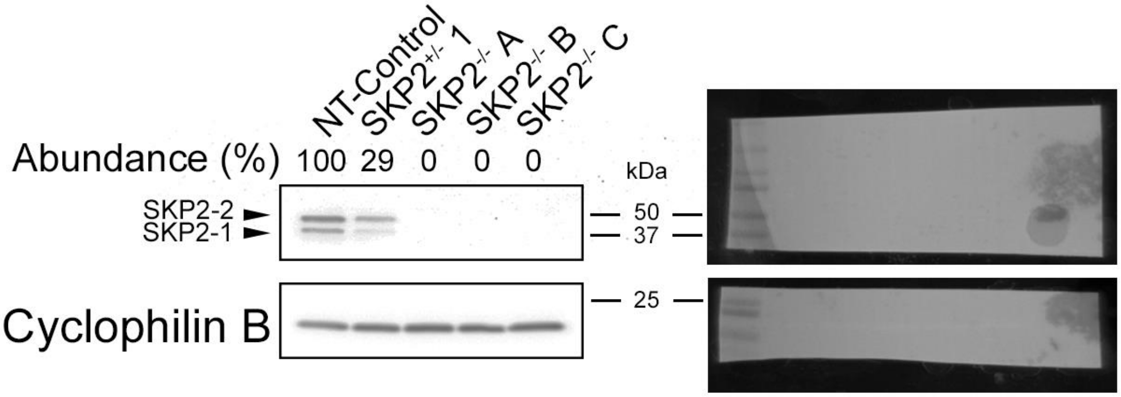

	SKP2	Cyclophilin B	Ratio S/C	Norm. ratio
**NT-Control**	48.84	33.15	1.47	100.00
SKP2$^{+/-}$ ***1***	18.25	43.01	0.42	28.80
SKP2$^{-/-}$ ***A***	0.06	51.41	0.00	0.08
SKP2$^{-/-}$ ***B***	0.02	45.29	0.00	0.03
SKP2$^{-/-}$ ***C***	0.04	52.89	0.00	0.06

Figure S6: Raw data for western blot in Figure S5.
Chemiluminescence (left) and visible light (right) images of the SKP2 and Cyclophilin B western blots shown in Figure S5A (p20). Black rectangles indicate cropped sections shown in Figure S5A. Densitometry analyses for SKP2 and Cyclophilin B were performed using Image J and are indicated. The ratio of SKP2/Cyclophilin B is shown for each lane, as are the normalized ratios, which are presented relative to NT-Control.

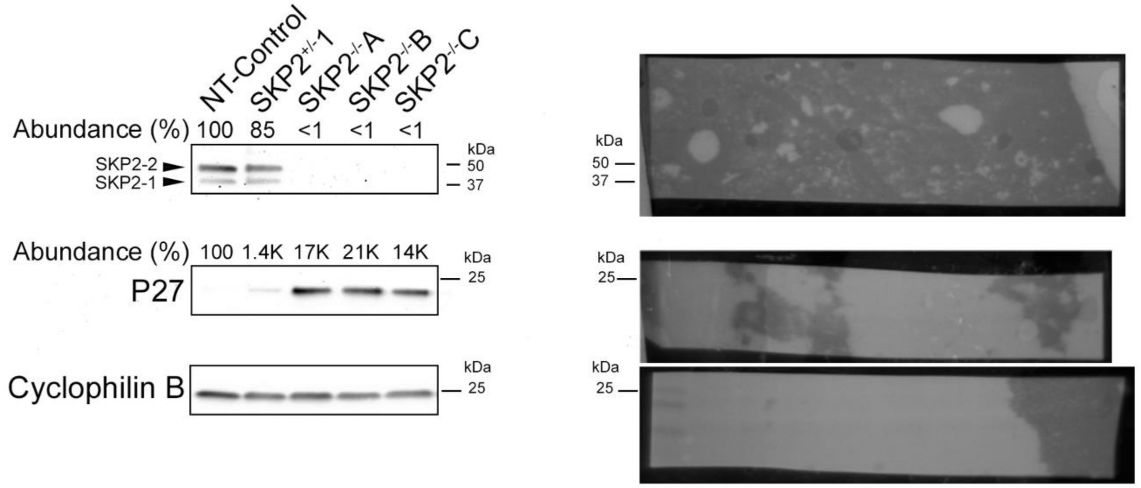

				Normalized Abundance		Relative Abundance	
	**SKP2**	**P27**	**Cyclophilin B**	**SKP2/CycloB**	**P27/CycloB**	**SKP2/CycloB**	**P27/CycloB**
**NT-Control**	35166.217	225.849	44352.459	0.792880886	0.005092142	100.0	100.0
***SKP2*$^{+/-}$1**	23707.146	2552.912	35284.338	0.671888644	0.072352555	84.7	1420.9
***SKP2*$^{-/-}$A**	91.607	34152.137	38924.51	0.002353453	0.877394141	0.3	17230.4
***SKP2*$^{-/-}$B**	122.314	31519.874	28734.752	0.004256658	1.096925214	0.5	21541.5
***SKP2*$^{-/-}$C**	169.849	24486.51	33329.874	0.005095999	0.734671544	0.6	14427.6

Figure S7. Semi-quantitative western blot depicting SKP2 and P27 abundance in *SKP2* clones.

Note that two bands representing the two SKP2 isoforms (SKP2-1 and SKP2-2; left labeling) are visible. Semi-quantitative analyses were performed whereby SKP2 and P27 abundance were first normalized to the respective loading control (Cyclophilin B) and are presented relative to NT-Control (100%). Densitometry analyses for SKP2, P27 and Cyclophilin B were performed using Fiji and are indicated. The relative abundance (%) of SKP2 and P27 are presented above each respective lane.

SUPPLEMENTARY TABLES

Table S1. Antibodies and Dilutions for Western Blot Analyses

Antibody	Source	Catalogue Number	Species	Dilution
**Primary Antibodies**				
SKP2	Invitrogen	32-33190	Mouse	1:500
P27	Abcam	ab32034	Rabbit	1:5000
Cyclophilin B	Abcam	ab16045	Rabbit	1: 150,000
**Secondary Antibodies**				
Goat α Rabbit HRP[A]	Jackson ImmunoResearch	111-035 144	Goat	1:10,000
Goat α Mouse HRP	Jackson ImmunoResearch	115-035-146	Goat	1:10,000

[A]HRP (Horseradish peroxidase)

Table S2. sgRNA Sequences and *SKP2* Target Sites.

sgRNA	Sequence[A]	Target Site
sgNT	5’-CGCGAUAGCGCGAAUAUAUU’3’	None
sg*SKP2*-1	5’-CCUGUCUGUGCCUCCCUGAGCUG-3’	*SKP2* Exon 3
sg*SKP2*-2	5’-UCUCUGGUGUUUGUAAGAGGUGG-3’	*SKP2* Exon 3

[A]Each sgRNA is composed of a variable 20 nucleotide sequence at its 3’ end (shown above), which either does not target any sequences in the human genome (sgNT) or targets a complementary region within *SKP2* (sg*SKP2*-1or sg*SKP2*-2). A constant 82 nucleotide sequence at the 5’ end of each sgRNA enables ribonucleoprotein complex formation with the Cas9: 5’UUUUUUCGUGGCUGAGCCACGGUGAAAAAGUUCAACUAUUGCCUGAUCGGAAUAAAAU UGAACGAUAAAGAUCGAGAUUUUG3’.

Table S3. Two-sample KS Tests Identify Significant Increases in Nuclear Area Distributions Following *SKP2* Silencing in HCT116

Condition	n[A]	*p*-value[B]	Significance[C]	D-statistic[D]
siControl	>1000	-	-	-
siSKP2-3	>1000	<0.0001	****	0.2429
siSKP2-4	>1000	<0.0001	****	0.2804
siSKP2-Pool	>1000	<0.0001	****	0.2385

[A]Number of nuclei analyzed.
[B]*p*-values calculated from two-sample KS tests for the listed condition relative to non-targeting silencing control.
[C]Significance: ****, p-value <0.0001.
[D]D-statistic (maximum deviation between the two distribution curves).

Table S4. MW Tests Fail to Identify Significant Increases in Micronucleus Formation Following *SKP2* Silencing in HCT116

Condition	n[A]	Mean Nucleus Count[B]	Mean MN Count[C]	Mean% MNF[D]	Median Fold Change in MNF[E]	*p*-value[F]	Sig.[G]
siControl	6	928	20.3	2.2	-	-	-
siSKP2-3	6	742	23.5	2.8	0.8	0.9372	ns
siSKP2-4	6	786	27.8	3.4	1.2	0.4848	ns
siSKP2-Pool	6	878	32.3	2.6	1.4	0.9372	ns

[A]Number of nuclei analyzed.
[B]Mean number of nuclei analyzed per well.
[C]Mean number of micronuclei counted per well.
[D]Mean percent MNF (calculated for each well as the MN count / nucleus count × 100).
[E]Median fold change in MNF relative to non-targeting control.
[F]p-values calculated from two-sample MW tests for the listed condition relative to non-targeting control at the corresponding timepoint.
[G]Significance: ns, p-value >0.05

Table S5. Student's t-tests Identify Significant Increases in the Frequency of Aberrant Chromosome Numbers in *SKP2* silenced HCT116 Cells

Condition	n[A]	p-value[B]	Significance[C]
siControl	300	-	-
siSKP2-3	300	0.0041	**
siSKP2-4	300	0.0048	**
siSKP2-Pool	300	0.0112	*

[A]Number of nuclei analyzed.
[B]*p*-values calculated from unpaired Student's T-Tests for the listed condition relative to non-targeting control.
[C]Significance: *, p-value < 0.05; **, p-value < 0.01

Table S6. Two-sample KS Tests Reveal Significant Increases in Nuclear Area Distributions Following *SKP2* Silencing in 1CT and A1309 Cells

Condition	n[A]	*p*-value[B]	Significance[C]	D-statistic[D]
		**1CT**		
siControl	>1000	-	-	-
siSKP2-3	>1000	<0.0001	****	0.2880
siSKP2-4	>1000	<0.0001	****	0.3659
siSKP2-Pool	>1000	<0.0001	****	0.2013
		**A1309**		
siControl	>1000	-	-	-
si*SKP2*-3	>1000	<0.0001	****	0.7318
si*SKP2*-4	>1000	<0.0001	****	0.3913
si*SKP2*-Pool	>1000	<0.0001	****	0.4706

[A]Number of nuclei analyzed.
[B]*p*-values calculated from two-sample KS tests for the listed condition relative to non-targeting silencing control.
[C]Significance: ****, p-value < 0.0001.
[D]D-statistic (maximum deviation between the two distribution curves).

Table S7. MW Tests Reveal Increases in Micronucleus Formation Following *SKP2* Silencing in 1CT and A1309 Cells.

Condition	n[A]	Mean Nucleus Count[B]	Mean MN Count[C]	Mean% MNF[D]	Median Fold Change in MNF[E]	*p*-value[F]	Sig.[G]
				**1CT**			
siControl	6	521	4.2	0.8	-	-	-
siSKP2-3	6	399	4.7	1.2	1.9	0.3095	ns
siSKP2-4	6	324	4.5	1.4	2.5	0.2403	ns
siSKP2-Pool	6	322	4.3	1.3	2.3	0.1797	ns
				**A1309**			
siControl	6	904	10.1	1.1	-		-
siSKP2-3	6	231	8.0	3.5	1.7	0.1797	ns
siSKP2-4	6	595	19.5	3.4	2.2	< 0.0001	****
siSKP2-Pool	6	484	18.3	3.9	2.6	0.0043	**

[A]Number of wells analyzed.
[B]Mean number of nuclei analyzed per well.
[C]Mean number of micronuclei counted per well.
[D]Mean percent MNF (calculated for each well as the MN count / nucleus count × 100).
[E]Median fold change in MNF relative to non-targeting control at the corresponding timepoint.
[F]p-values calculated from two-sample M-W tests for the listed condition relative to non-targeting silencing control at the corresponding timepoint.
[G]Significance: ns, p-value > 0.05; **, p-value <0.01; ****, p-value <0.0001

Table S8. Student's t-tests Identify Significant Increases in the Frequency of Aberrant Chromosome Numbers in *SKP2* silenced 1CT and A1309 Cells

Condition	n[A]	p-value[B]	Significance[C]
		**1CT**	
siControl	300	-	-
siSKP2-3	300	0.1028	ns
siSKP2-4	300	0.0374	*
siSKP2-Pool	300	0.1123	ns
		**A1309**	
siControl	300	-	-
siSKP2-3	300	<0.0001	***
siSKP2-4	300	0.0293	*
siSKP2-Pool	300	0.0646	ns

[A]Number of nuclei analyzed.

[B]*p*-values calculated from two-sample KS tests for the listed condition relative to non-targeting silencing control.

[C]Significance: ns, p-value > 0.05; *, p-value < 0.05; ***, p-value < 0.001

Table S9. Two-sample KS Tests Reveal Significant Changes in Nuclear Area Distributions in A1309 *SKP2*$^{+/-}$ and *SKP2*$^{-/-}$ Clones Over Time

Condition	n[A]	*p*-value[B]	Significance[C]	D-statistic[D]
**p0**				
NT-Control	>1000	-	-	-
SKP2$^{+/-}$ 1	>1000	<0.0001	****	0.1536
SKP2$^{-/-}$ A	>1000	<0.0001	****	0.1660
SKP2$^{-/-}$ B	>1000	<0.0001	****	0.2786
SKP2$^{-/-}$ C	>1000	<0.0001	****	0.1762
**p4**				
NT-Control	>1000	-	-	-
SKP2$^{+/-}$ 1	>1000	<0.0001	****	0.1462
SKP2$^{-/-}$ A	>1000	<0.0001	***	0.0465
SKP2$^{-/-}$ B	>1000	<0.0001	****	0.1187
SKP2$^{-/-}$ C	>1000	<0.0001	****	0.0518
**p8**				
NT-Control	>1000	-	-	-
SKP2$^{+/-}$ 1	>1000	<0.0001	****	0.0513
SKP2$^{-/-}$ A	>1000	<0.0001	****	0.1129
SKP2$^{-/-}$ B	>1000	<0.0001	****	0.2564
SKP2$^{-/-}$ C	>1000	<0.0001	****	0.0731
**p12**				
NT-Control	>1000	-	-	-
SKP2$^{+/-}$ 1	>1000	<0.0001	****	0.1109
SKP2$^{-/-}$ A	>1000	<0.0001	****	0.0913
SKP2$^{-/-}$ B	>1000	<0.0001	****	0.2440
SKP2$^{-/-}$ C	>1000	0.0009	***	0.0416
**p16**				
NT-Control	>1000	-	-	-
SKP2$^{+/-}$ 1	>1000	<0.0001	****	0.1040
SKP2$^{-/-}$ A	>1000	<0.0001	****	0.2082
SKP2$^{-/-}$ B	>1000	<0.0001	****	0.1698
SKP2$^{-/-}$ C	>1000	<0.0001	****	0.0629
**p20**				
NT-Control	>1000	-	-	-
SKP2$^{+/-}$ 1	>1000	0.2934	ns	0.0212
SKP2$^{-/-}$ A	>1000	<0.0001	****	0.1545
SKP2$^{-/-}$ B	>1000	<0.0001	****	0.2029
SKP2$^{-/-}$ C	>1000	<0.0001	****	0.1217

[A]Number of nuclei analyzed.
[B]*p*-values calculated from two-sample KS tests for the listed condition relative to non-targeting control.
[C]Significance: ns, p-value > 0.05; ***, p-value < 0.001; ****, p-value < 0.0001.
[D]D-statistic (maximum deviation between the two distribution curves).

Table S10. **Statistical Assessment of Micronuclei within *SKP2*$^{+/-}$ and *SKP2*$^{-/-}$ Models Over Time**

Condition	n[A]	Mean Nucleus Count[B]	Mean MN Count[C]	Mean% MNF[D]	Median Fold Change in MNF[E]	*p*-value[F]	Sig.[G]
**p0**							
NT-Control	6	624	17.0	2.7	-	-	-
SKP2$^{+/-}$ 1	6	603	30.2	5.0	1.6	0.0087	**
SKP2$^{-/-}$ A	6	530	19.7	3.7	1.1	0.3939	ns
SKP2$^{-/-}$ B	6	464	37.2	8.0	3.0	0.0087	**
SKP2$^{-/-}$ C	6	506	20.5	4.1	1.2	0.2403	ns
**p4**							
NT-Control	6	647	1.3	0.2	-	-	-
SKP2$^{+/-}$ 1	6	474	5.8	1.1	6.8	0.0043	**
SKP2$^{-/-}$ A	6	681	23.0	3.4	26.0	0.0022	**
SKP2$^{-/-}$ B	6	680	36.2	5.3	25.8	0.0022	**
SKP2$^{-/-}$ C	6	714	14.7	2.1	13.9	0.0022	**
**p8**							
NT-Control	6	714	6.3	0.9	-	-	-
SKP2$^{+/-}$ 1	6	713	17.0	2.5	2.2	0.0022	**
SKP2$^{-/-}$ A	6	622	13	2.1	2.4	0.0043	**
SKP2$^{-/-}$ B	6	569	22.5	4.4	5.3	0.0022	**
SKP2$^{-/-}$ C	6	610	19	3.1	3.4	0.0043	**
**p12**							
NT-Control	6	761	3.7	0.5	-	-	-
SKP2$^{+/-}$ 1	6	591	6.7	1.1	2.3	0.0411	*
SKP2$^{-/-}$ A	6	473	3.5	0.8	1.0	0.8182	ns
SKP2$^{-/-}$ B	6	535	12.7	2.5	4.4	0.0022	**
SKP2$^{-/-}$ C	6	720	9.8	1.3	2.5	0.0649	ns
**p16**							
NT-Control	6	806	7.7	1.0	-	-	-
SKP2$^{+/-}$ 1	6	748	18.0	2.4	2.8	0.0022	**
SKP2$^{-/-}$ A	6	516	10.7	2.1	2.0	0.0931	ns
SKP2$^{-/-}$ B	6	766	22.0	2.9	3.3	0.0022	**
SKP2$^{-/-}$ C	6	786	15.7	2.0	2.2	0.0022	**
**p20**							
NT-Control	6	727	8.0	1.1	-	-	-
SKP2$^{+/-}$ 1	6	720	12.2	1.7	1.6	0.0931	ns
SKP2$^{-/-}$ A	6	533	12.5	2.4	2.1	0.0260	*
SKP2$^{-/-}$ B	6	627	19.2	3.1	3.0	0.0022	**
SKP2$^{-/-}$ C	6	689	10.2	1.5	1.3	0.0931	ns

[A]Number of wells analyzed.
[B]Mean number of nuclei analyzed per well.
[C]Mean number of micronuclei counted per well.
[D]Mean percent MNF (calculated for each well as the MN count / nucleus count × 100).
[E]Median fold change in MNF relative to non-targeting control at the corresponding timepoint.
[F]p-values calculated from two-sample MW tests for the listed condition relative to non-targeting control at the corresponding timepoint.
[G]Significance: ns, p-value > 0.05; *, p-value < 0.05; **, p-value < 0.01.

Table S11. Statistical Assessment of Chromosome Numbers Within *SKP2*$^{+/-}$ and *SKP2*$^{-/-}$ Clones Over Time

Condition	n[A]	Fold Change in Aberrant Spreads	*p*-value[B]	Significance[C]	D-statistic[D]
**p0**					
NT-Control	100	-	-	-	-
SKP2$^{+/-}$ 1	100	4.9	0.0023	**	0.2600
SKP2$^{-/-}$ A	100	3.8	0.0541	ns	0.1900
SKP2$^{-/-}$ B	100	4.0	0.0243	*	0.2100
SKP2$^{-/-}$ C	100	4.4	<0.0001	****	0.3500
**p4**					
NT-Control	100	-	-	-	-
SKP2$^{+/-}$ 1	100	2.7	0.2106	ns	0.1500
SKP2$^{-/-}$ A	100	2.3	0.0541	ns	0.1900
SKP2$^{-/-}$ B	100	2.3	0.0783	ns	0.1800
SKP2$^{-/-}$ C	100	2.9	0.0366	*	0.2000
**p8**					
NT-Control	100	-	-	-	-
SKP2$^{+/-}$ 1	100	1.4	0.9671	ns	0.0700
SKP2$^{-/-}$ A	100	0.7	>0.9999	ns	0.0300
SKP2$^{-/-}$ B	100	1.9	0.9996	ns	0.0500
SKP2$^{-/-}$ C	100	1.5	0.9996	ns	0.0500
**p12**					
NT-Control	100	-	-	-	-
SKP2$^{+/-}$ 1	100	0.5	0.9938	ns	0.0600
SKP2$^{-/-}$ A	100	1.8	0.9671	ns	0.0700
SKP2$^{-/-}$ B	100	1.6	0.6994	ns	0.1000
SKP2$^{-/-}$ C	100	1.6	0.5806	ns	0.1100
**p16**					
NT-Control	100	-	-	-	-
SKP2$^{+/-}$ 1	100	3.1	0.0158	*	0.2200
SKP2$^{-/-}$ A	100	1.8	0.6994	ns	0.1000
SKP2$^{-/-}$ B	100	2.4	0.0783	ns	0.1800
SKP2$^{-/-}$ C	100	2.9	0.0541	ns	0.1900
**p20**					
NT-Control	100	-	-	-	-
SKP2$^{+/-}$ 1	100	1.5	0.9938	ns	0.0600
SKP2$^{-/-}$ A	100	1.8	0.0541	ns	0.1900
SKP2$^{-/-}$ B	100	1.0	>0.9999	ns	0.0300
SKP2$^{-/-}$ C	100	0.9	>0.9999	ns	0.0400

[A]Number of MCS analyzed.
[B]*p*-values calculated from two-sample KS tests for the listed condition relative to non-targeting control.
[C]Significance: ns, p-value > 0.05; *, p-value < 0.05; **, p-value < 0.01; ****, p-value < 0.0001.

[D] D-statistic (maximum deviation between the two distribution curves).
